# miR-22-5p regulates the self-renewal of spermatogonial stem cells by targeting EZH2

**DOI:** 10.1515/med-2022-0429

**Published:** 2022-03-17

**Authors:** Wenqiang Lv, Mei Yu, Yilin Su

**Affiliations:** Department of Pediatric Surgery, The First Affiliated Hospital of USTC, Division of Life Sciences and Medicine, University of Science and Technology of China, Hefei 230001, Anhui, China; Department of Pediatrician, Binhu District of Hefei First People’s Hospital (The Third Affiliated Hospital of Anhui Medical University), Hefei 230000, Anhui, China

**Keywords:** cryptorchidism, spermatogonial stem cells, miR-22-5p

## Abstract

MiRNAs play an important role in spermatogonial stem cells (SSCs). The purpose of this study was to investigate the basic function of miR-22-5p in cryptorchidism. The results of RT-PCR, western blot, and immunohistochemistry showed that miR-22-5p was increased while EZH2 decreased in the testicular tissues of patients with cryptorchidism. Overexpression of miR-22-5p inhibited the proliferation of SSCs, increased cell apoptosis rate, and reduced expression of SSC marker proteins (GDNF and DAZL); however, knockout of miR-22-5p has the opposite effect. The Luciferase reporter gene assays demonstrated that EZH2 is a direct target of miR-22-5p. Moreover, EZH2 overexpression could reverse the effect of miR-22-5p mimic on SSCs’ proliferation, apoptosis, and expression of SSC marker proteins. Our results demonstrated that miR-22-5p regulates SSCs’ self-renewal by targeting EZH2, which indicated that miR-22-5p may serve as a biological marker for the treatment of infertility caused by cryptorchidism.

## 1 Introduction

Cryptorchidism, also known as undescended testis (UDT), is the most common birth defect involving male genitalia. About 3% of full-term and 30% of premature male infants are born with one or both testicles undescended [1]. Affected by hormone, temperature, genes, and other factors, the testes that did not fall into the scrotum appear as spermatogenesis obstruction and germ cell apoptosis, which is a common cause of male infertility [2,3]. Understanding the molecular mechanism of spermatogenesis disorder and germ cell apoptosis is helpful to better understand germ cell differentiation and find a new method for the treatment of infertility caused by cryptorchidism.

Spermatogonial stem cells (SSCs) are the only adult stem cells that can transmit genetic information to their offspring, which is the basis of spermatogenesis [4,5]. SSCs maintain a stable number of SSCs and sperm in males through self-renewal and differentiation. Excessive proliferation of SSCs will lead to excessive accumulation of SSCs and affect normal spermatogenesis. On the contrary, it will lead to the depletion of SSCs [6]. Therefore, maintaining the balance between self-renewal and differentiation of SSCs is an important prerequisite for the sustained sperm production of the testis.

MicroRNAs (miRNAs) play a key role in the control of gene expression in a wide array of tissue systems, where their functions include the regulation of self-renewal, cellular differentiation, proliferation, and apoptosis [7,8]. Studies have shown that miRNAs play an important role in spermatogenesis [9,10]. Li et al. [11] have reported that miR-130a could negatively regulate AR expression in mouse Sertoli cells, which further causes defects in spermatogenesis. In addition, miR-322 [12], miR-30a-5p [13], miR-31-5p [14], miR-122-5p [15], and so on were reported to regulate self-renewal, differentiation, proliferation and apoptosis of SSCs. Previous studies have shown that miR-22-5p is abnormally expressed in acute myocardial infarction [16,17], cancer [18,19], Alzheimer’s disease [20], and other diseases [21], which could be considered promising novel diagnostic biomarkers for these diseases. Using microarray analysis, Moritoki et al. [22] compared total miRNA expression in unilateral undescended testes with that in contralateral descended and normal testes and found that miR-22-5p was significantly highly expressed in testicular tissues of cryptorchidism patients (FD = 2.53, *p* < 0.05), suggesting that miR-22-5p may be involved in the regulation of cryptorchidism disorder.

Enhancer of zeste homolog 2 (EZH2) is a histone H3 lysine 27 (H3K27) methyltransferase that plays a vital role in spermatogenesis and self-renewal of SSCs, [23,24]. The predicted analysis of the target gene of miR-22-5p showed that there was a binding site between miR-22-5p and EZH2 3′UTR. Therefore, we speculate that miR-22-5p may regulate the self-renewal of SSCs by regulating the expression of EZH2.

## 2 Materials and methods

### 2.1 Tissue samples collection

Human testicular tissues were obtained from the First Affiliated Hospital of the University of Science and Technology of China (USTC, Hefei, China). A total of 10 samples of testicular tissues from patients with cryptorchidism and another 10 samples of testicular tissues from people with normal fertility were collected for comparison. The normal testicular tissues were collected from patients during surgical treatment or biopsy. Johnsen score was used to objectively evaluate spermatogenesis in the two groups. In this study, the Johnsen score in patients with obstructive azoospermia was 8–9, and that in patients with cryptorchidism was only 3–4, indicating that there was significant spermatogenesis disorder in testicular tissues of patients with cryptorchidism compared with obstructive azoospermia.

Clinical characteristics of the cryptorchidism patients were as follows: among the 10 patients, there were 3 cases of left cryptorchidism (30.00%), 6 cases of right cryptorchidism (60.00%), and 1 case of bilateral cryptorchidism (10.00%); one case was complicated with penile malformation, accounting for 10.00%; testicular location: 4 cases (40.00%) in the abdominal cavity and 6 cases (60.00%) in the inguinal area. This study was approved by the Ethics Committee of the First Affiliated Hospital of USTC. All participants signed informed consent.

### 2.2 RT-PCR analysis

Extraction of total RNA in testicular tissues or cells was performed using the TRIzol reagent (Invitrogen). RNAs were subjected to reverse transcription. The extracted cDNA was applied for PCR using the SYBR-Green method. Primer sequences are shown in Table 1. The stem-loop RT-PCR was used to perform the qPCR of miR-22-5p. Complementary DNA was synthesized from RNA with the FastQuant RT Kit according to the manufacturer’s protocol. The primer of miR-22-5p used for cDNA synthesis is shown in Table 1. Real-time PCR was performed with the SuperReal PreMix Plus (SYBR Green) Kit in an ABI7500 Real Time PCR System (Applied Biosystems; Thermo Fisher Scientific, Inc.). The expression level of mature miR-22-5p or mRNA was normalized to U6 or GAPDH, respectively. Relative expression levels were calculated using DataAssist software (Applied Biosystems)using the formula 2^−ΔΔCt^.

**Table 1:** Primer sequences used in miRNA reverse transcription and PCR

Name	Primer (5′-3′)
Reverse transcription primers	
miR-22-5p	GTCGTATCCAGTGCGTGTCGTGGAGTCGGCAATTGCA
	CTGGATACGACTAAAGC
PCR primers	
miR-22-5p	F: GAGCTGCACTGACCAGTAGG
	R: GTGCTGGCAGATGGATCACT
U6	F: CTCGCTTCGGCAGCACA
	R: AACGCTTCACGAATTTGCGT
EZH2	F: CGGGGTACCGAGTCATACTTGTGAAG
	R: GCACTCGAGCCTGTTTTTGTTTGATG
GAPDH	F: GCACCGTCAAGGCTGAGAAC
	R: TGGTGAAGACGCCAGTGGA

### 2.3 Western blot analysis

The testicular tissues or human SSCs with the treatment of miRNA oligonucleotides/overexpression plasmid were lysed with RIPA buffer. The concentrations of proteins were measured by the BCA kit. Thirty micrograms of cell lysate from each cell sample were used for SDS-PAGE (Bio-Rad). Then, the proteins were transferred into PVDF membranes (Roche, Germany) and blocked with 5 % non-fat dry milk (Carnation, CA). Subsequently, the samples were incubated with primary antibodies against EZH2 (ab191250, Abcam), GDNF (ab176564, Abcam), DAZL (ab34139, Abcam), Caspase-3 (ab32042, Abcam), Bax (ab32503, Abcam), and Bcl-2 (ab32124, Abcam) overnight at 4°C. The nitrocellulose membrane was incubated for 2 h after adding appropriate secondary antibodies (HRP-conjugated goat anti-rabbit) (Abcam). Finally, the expression of proteins was evaluated using enhanced chemiluminescence.

### 2.4 Immunohistochemical analysis

The testicular tissue sections were heated in pH 6.0 sodium citrate buffer and then dipped in deionized water containing 3% H_2_O_2_ to inhibit endogenous peroxidase activity. The sections were incubated with an EZH2 specific antibody (ab191250, Abcam) and HRP-labelled secondary antibody, respectively. Finally, the sections were stained with diaminobenzidine and counterstained with Harris’s hematoxylin.

### 2.5 Human SSC culture and transfection

The human SSC line was cultured with DMEM/F12 supplemented with 10% FBS and 100 units/mL penicillin and streptomycin (Invitrogen). The cells were passaged every 3-4 days using 0.05% trypsin (Invitrogen) and 0.53 mM EDTA (Invitrogen), and they were maintained at 34°C in a humidified 5% CO_2_ incubator. The EZH2 overexpressing plasmid was purchased by Ribobio (Guangzhou, China). The miR-22-5p mimic/inhibitor was synthesized by Ribobio (Guangzhou, China). Transfection of RNA mimic/inhibitor was conducted with Oligofectamine (Invitrogen) according to the manufacturer’s protocol. Plasmid transfection was conducted with Lipofectamine 2000 (Invitrogen) according to the manufacturer’s protocol.

### 2.6 EdU staining assay for cell proliferation

Transfected cells were spread on a round coverslip and incubated with 100 mM EdU (EdU Assay Kit, Beyotime Biotechnology). Cells were then washed with PBS, fixed with 4% PFA, and washed with 2 mg/mL glycine for 5 min. Next, cells were permeabilized in PBS containing 0.5% Triton X-100 for 20 min after removing the glycine solution. After washing with PBS, cell nuclei were stained with Hoechst (Abcam, 1:2,000). The coverslip was then sealed with an antifade mounting medium, and a laser scanning confocal microscope (Zeiss, LSM700) was used to photograph the samples using the same conditions.

### 2.7 Flow cytometry analysis for cell apoptosis

Human SSCs were seeded at a density of 5 × 10^4^ cells/well in 12-well plates, and cells were collected on day 3 after transfection. Apoptosis in the human SSC line was measured using the Annexin V and PI apoptosis detection kit and flow cytometry according to the manufacturer’s instructions.

### 2.8 Luciferase reporter assays

EZH23′UTR including the predicted binding site of miR-22-5p (wt) or a site-directed gene mutated miR-22-5p-binding site (mut) was inserted downstream of the firefly luciferase gene of the psiCHECK2 vector (Promega). The wt or mut vector was co-transfected into SSCs with miR-22-5p mimic or mimic NC in 24-well plates. After 48 h, the cells were harvested and assayed by a Dual Luciferase Assay (Promega) following the manufacturer’s protocol.

### 2.9 Statistical analysis

The data are presented as the mean  ±  standard deviation (SD) and analyzed with GraphPad Prism 7.0 using one-way ANOVA and Tukey *post-hoc* test. The statistical significance was set at 0.05 (*p*  < 0.05).

## 3 Results

### 3.1 The expression of miR-22-5p was increased in the testicular tissues of patients with cryptorchidism.

**Figure 1:**
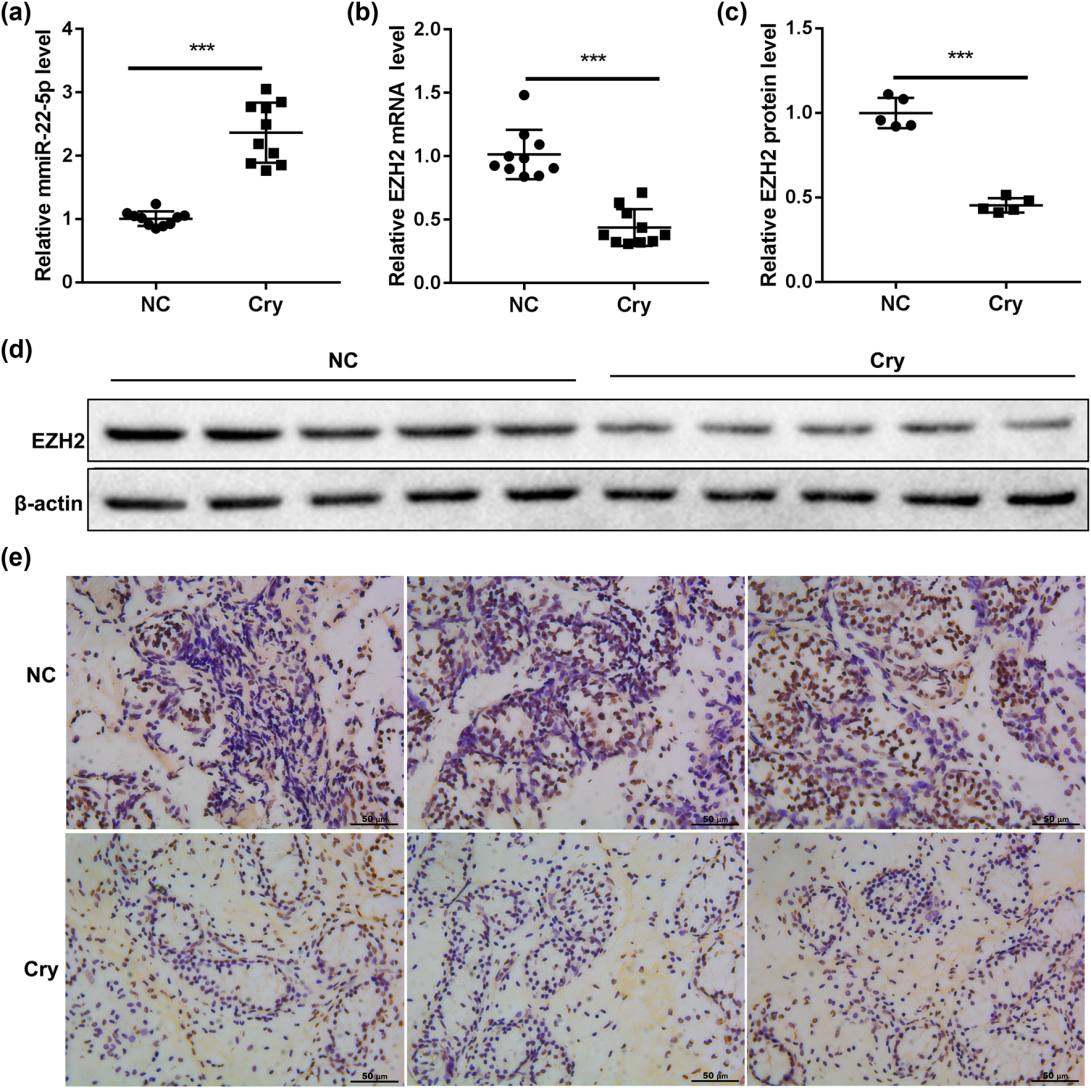
miR-22-5p was significantly upregulated in the testicular tissues of patients with cryptorchidism. Testicular tissues of cryptorchidism patients (cry group) and normal testicular tissues of fertile participants (NC group) were collected. (a) The expression of miR-22-5p was detected by QRT-PCR. (b–e) The expression of EZH2 in the testicular tissues was detected by QRT-PCR, western blot, and immunohistochemistry (scale bar = 50 µm). ***p < 0.001 vs NC group.

To investigate whether miR-22-5p functioned in the testicular tissues of patients with cryptorchidism, ten samples of testicular tissues from patients with cryptorchidism (cry group) and another ten samples of testicular tissues from people with normal fertility (NC group) were used for comparison. The RT-PCR results showed that the expression of miR-22-5p in the cry group was significantly higher than that in the NC group (Figure 1a), but the mRNA and protein expression of EZH2 were markedly reduced in the cry group (Figure 1b–d). In addition, the IHC results showed that there are more brown yellow, or brown dots in the NC group, indicating that EZH2 protein in the cry group is significantly lower than that of the NC group (Figure 1e). These data showed that miR-22-5p expression was increased while EZH2 expression was decreased in the testicular tissues of patients with cryptorchidism.

### 3.2 miR-22-5p regulates SSCs’ self-renewal

**Figure 2:**
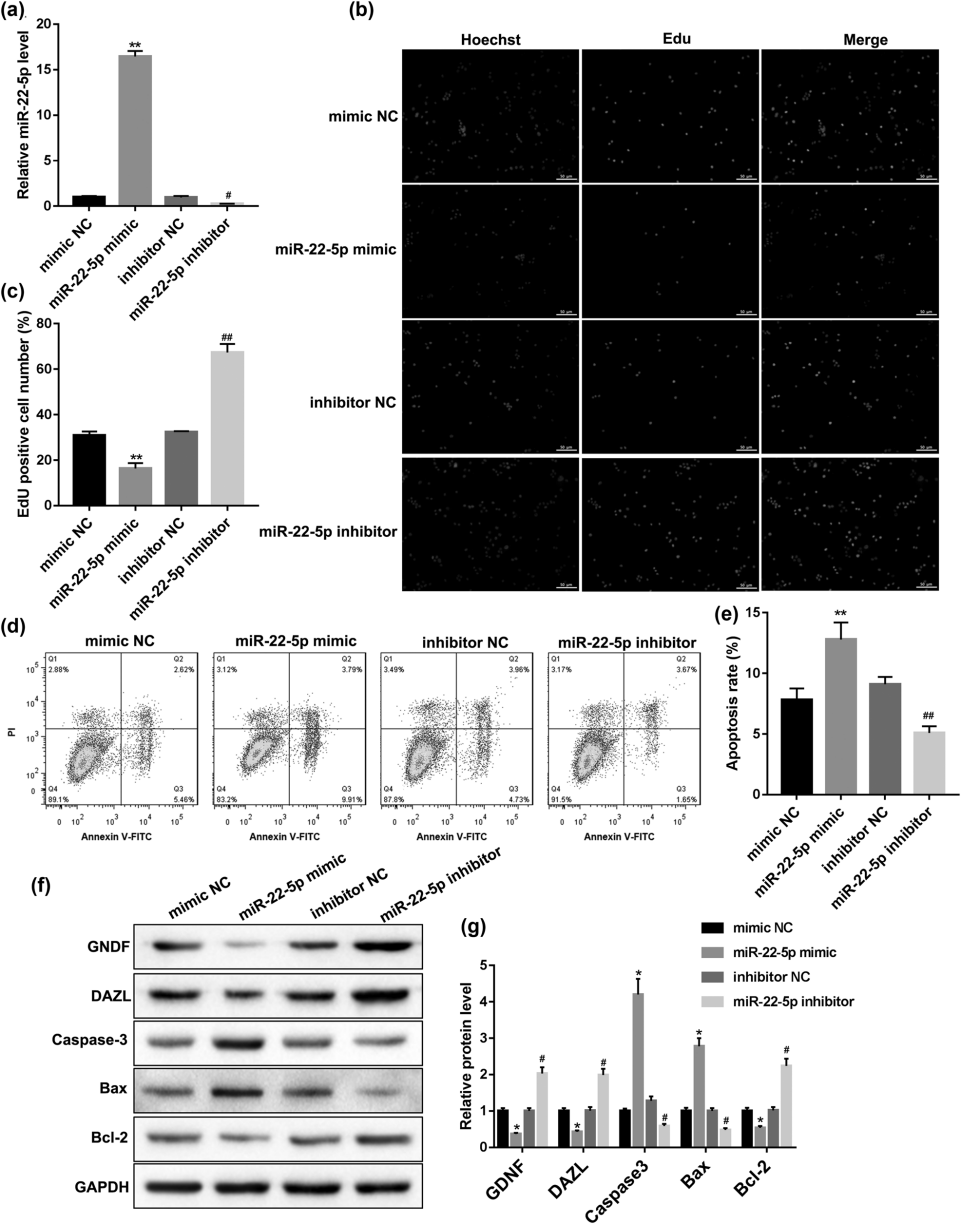
Effect of miR-22-5p on SSCs. Human SSCs were transfected with the miR-22-5p mimic or miR-22-5p inhibitor, respectively. (a) The miR-22-5p overexpression and interference efficiency were detected by QRT-PCR. (b and c) Cell proliferation was measured by EdU staining. (d and e) Cell apoptosis was analyzed by Annexin V/PI staining. (f and g) The expression of SSC markers (GDNF and DAZL) and apoptosis-related proteins (Caspase-3, Bax, and Bcl-2) were detected by western blot. *p < 0.05, **p < 0.01 vs mimic NC; #*p* < 0.05, ##*p* < 0.01 vs inhibitor NC.

Knowing the abnormal expression of miR-22-5p in the testicular tissues of patients with cryptorchidism, next, to investigate the effect of miR-22-5p on SSCs’ self-renewal, human SSCs were transfected with miR-22-5p mimic or miR-22-5p inhibitor to overexpress or knock outmiR-22-5p, respectively. As shown in Figure 2a, the miR-22-5p overexpression, and knockout efficiency were detected by QRT-PCR. Then, the EdU assay showed that miR-22-5p mimic the reduced EdU positive cell number, while miR-22-5p inhibitor increased the EdU positive cell number (Figure 2b and c), indicating that miR-22-5p had a significant regulatory effect on the proliferation of SSCs. Meanwhile, the flow cytometry analysis showed that miR-22-5p mimic transfection increased cell apoptosis rate, while miR-22-5p inhibitor transfection decreased the cell apoptosis rate (Figure 2d and e). Furthermore, the western blot results showed that the trend of apoptotic proteins expression (Caspase-3, Bax and Bcl-2) was consistent with that of flow cytometry (Figure 2f and g), indicating that miR-22-5p had a significant regulatory effect on the apoptosis of SSCs. In addition, the expression of SSC marker proteins (GDNF and DAZL) were decreased by miR-22-5p mimic but increased by the miR-22-5p inhibitor, implying the effect of miR-22-5p on SSCs’ self-renewal.

### 3.3 EZH2 is a direct target of miR-22-5p

**Figure 3:**
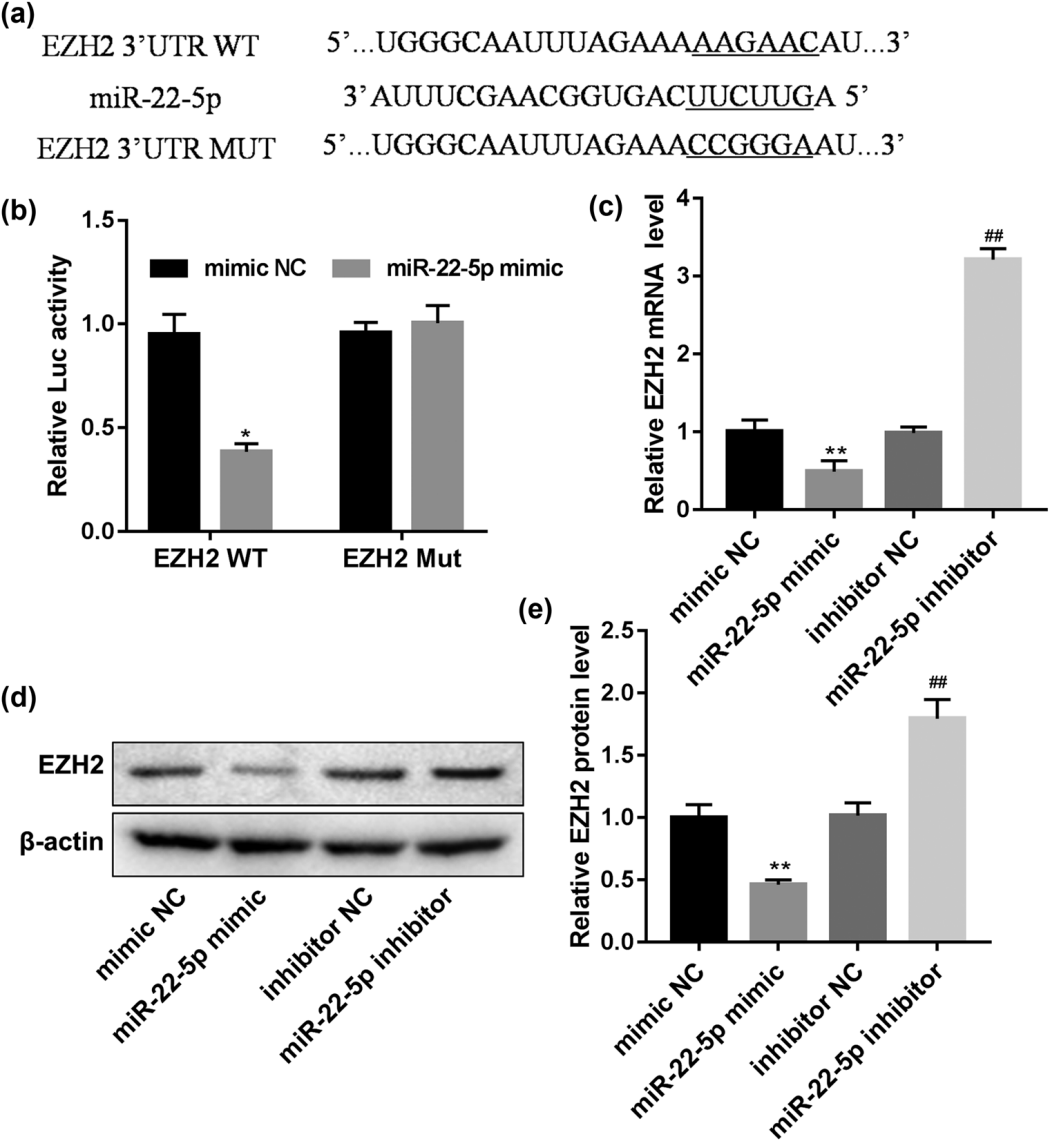
miR-22-5p directly targets EZH2. (a) The target region of the EZH2 3′UTR for miR-22-5p and the mutant type of EZH2 3′UTR. (b) Effects of miR-22-5p on the activity of firefly luciferase reporters containing either wild-type (Wt) or mutant-type (Mut) 3′UTR were assessed by luciferase reporter gene assays. (c–e) Effects of miR-22-5p on EZH2 expression levels were examined by qRT-PCR and western blot analyses. *p < 0.05, **p < 0.01 vs mimic NC; ##*p* < 0.01 vs inhibitor NC.

To explore the mechanism of miR-22-5p promoting self-renewal of SSCs, TargetScan was used to predict the possible target genes. Then, we found that EZH2 is a target gene of miR-22-5p (Figure 3a). Subsequently, the luciferase reporter gene assays demonstrated that miR-22-5p can bind to the EZH2 mRNA 3′ UTR (Figure 3b). Furthermore, the mRNA and protein expression of EZH2 were remarkably reduced by the miR-22-5p mimic and increased by the miR-22-5p inhibitor (Figure 3c–e), which suggested that EZH2 is a direct target of miR-22-5p.

### 3.4 miR-22-5p regulates SSCs’ self-renewal by targeting EZH2

**Figure 4:**
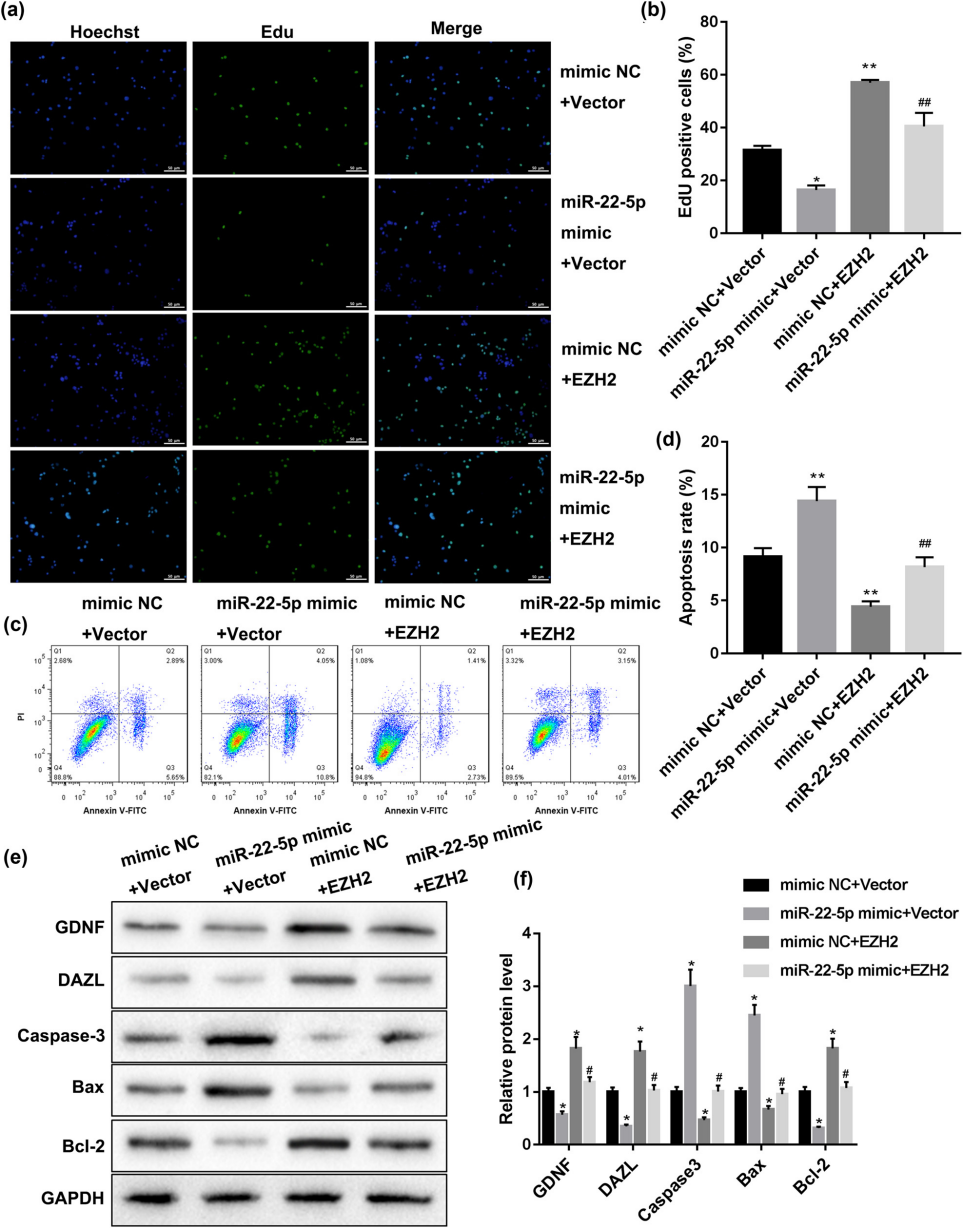
miR-22-5p regulates SSCs’ self-renewal by targeting EZH2. Human SSCs were co-transfected with miR-22-5p mimics and the EZH2 overexpression plasmid. (a and b) Cell proliferation was measured by EdU staining. (c and d) Cell apoptosis was analyzed by Annexin V/PI staining. (e and f) The expression of SSC markers (GDNF and DAZL) and apoptosis-related proteins (Caspase-3, Bax and Bcl-2) were detected by western blot. *p < 0.05, **p < 0.01 vs mimic NC + vector; #*p* < 0.05, ##*p* < 0.01 vs miR-22-5p mimic + vector.

Finally, to explore whether miR-22-5p participates in spermatogenesis by regulating EZH2, we examined the reverse effect of EZH2 overexpression on the regulation of miR-22-5p on SSCs’ self-renewal. As shown in Figure 4a and b, the miR-22-5p mimic reduced the EdU positive cell number, the EZH2 overexpression plasmid increased the EdU positive cell number, and EZH2 overexpression could reverse the effect of the miR-22-5p mimic on SSCs’ proliferation. The flow cytometry results showed that miR-22-5p mimic increased SSCs’ apoptosis rate, the EZH2 overexpression plasmid decreased SSCs’ apoptosis rate, and EZH2 overexpression could reverse the effect of miR-22-5p mimic on SSCs’ apoptosis (Figure 4c and d). EZH2 overexpression could reverse the effect of miR-22-5p mimic on the expression of apoptosis-related proteins (Caspase-3, Bax, and Bcl-2) (Figure 4e and f). Besides, the expression of SSCs’ marker proteins (GDNF and DAZL) was decreased by miR-22-5p mimic, increased by the EZH2 overexpression plasmid, and EZH2 overexpression could reverse the effect of miR-22-5p mimic on the expression of GDNF and DAZL. These data demonstrated that miR-22-5p regulates SSCs’ self-renewal by targeting EZH2.

## 4 Discussion

Spermatogonia, especially SSCs, is the key factor to maintain spermatogenesis [6]. Spermatogenesis is a process of proliferation and differentiation of male germ cells, in which post-transcriptional regulation is indispensable. As well known, miRNAs are one of the most common genes involved in post-transcriptional regulation [7,8]. Up to now, many miRNAs have been reported to be upregulated in the testicular tissues of patients with cryptorchidism [9,10]. Moritoki et al. [22] found that miR-22-5p was significantly highly expressed in unilateral undescended testes than that in normal testes in a rat model of cryptorchidism. In this study, we found that miR-22-5p was significantly upregulated in the testicular tissues of patients with cryptorchidism, which is consistent with the previous report.

Recent studies have reported that miR-22-5p is involved in hair follicle stem cell proliferation and differentiation [25]. Here, we propose that miR-22-5p may serve as a novel target for the proliferation and differentiation of SSCs. Subsequently, the effect of miR-22-5p on proliferation and differentiation of SSCs was studied by transfecting with the miR-22-5p mimic or miR-22-5p inhibitor. Our result reveals that the miR-22-5p mimic inhibited the proliferation of SSCs, increased cell apoptosis rate, and reduced the expression of SSC marker proteins (GDNF and DAZL); however, the miR-22-5p inhibitor has the opposite effect. The data suggested that miR-22-5p regulates SSCs’ self-renewal.

EZH2 is required for stable embryonic stem cells (ESCs) self-renewal by reducing H3K27me3 [26,27], and its expression in the testes has been previously reported [28]. Here, we found that EZH2 mRNA and protein expression were both significantly decreased in the testicular tissues of patients with cryptorchidism. According to many literature studies, EZH2 is the downstream target gene of multiple miRNAs [29]. Through TargetScan prediction, miR-22-5p could bind to the EZH2 mRNA 3′ UTR. Then, we confirmed their target binding relationship by the luciferase reporter gene assay. And further tests showed that the mRNA and protein expression of EZH2 were remarkably reduced by the miR-22-5p mimic and increased by miR-22-5p inhibitor, which suggested that EZH2 is a direct target of miR-22-5p. A recent study showed that EZH2 plays a pivotal role in the self-renewal of goat SSCs, and the knockdown of EZH2 might impair spermatogenesis in goats [24]. Here, we also demonstrated that EZH2 overexpression could reverse the effect of miR-22-5p mimic on SSCs’ proliferation, apoptosis, and SSCs; marker proteins expression, implying that miR-22-5p regulates SSCs’ self-renewal by targeting EZH2.

In conclusion, our results suggest that upregulation of miR-22-5p affects the self-renewal of human SSCs by targeting EZH2, which plays a key role in spermatogenesis, including the inhibition of cell proliferation, an increase of apoptosis, and changes of related gene expression. Our research provides new insights into the mechanism of male infertility caused by cryptorchidism. We propose that miR-22-5p may serve as a novel target for the treatment of infertility caused by cryptorchidism.
